# Metabolomic Analysis of Sea Cucumber Ovum Hydrolysates in Cyclophosphamide-Induced Premature Ovarian Failure

**DOI:** 10.3390/foods14213605

**Published:** 2025-10-23

**Authors:** Xinxin Wang, Leilei Sun, Mingbo Li, Shunmin Gong, Shijia Huang, Jiarun Gao, Yu Zhang, Liqin Sun

**Affiliations:** Yantai Key Laboratory of Characteristic Agricultural Bioresource Conservation & Germplasm Innovative Utilization, School of Life Sciences, Yantai University, Yantai 264005, China; 13562789830@163.com (X.W.); leilei.198966@163.com (L.S.); limingbo1711@163.com (M.L.); g18807041394@163.com (S.G.); sjhuang01@163.com (S.H.); 15863805335@163.com (J.G.)

**Keywords:** sea cucumber ovum hydrolysates, premature ovarian failure, metabolomics

## Abstract

Sea cucumber ovum are high-value compounds that remain after the processing of sea cucumbers, and their optimal utilization has long posed a challenge. In this research, we systematically examined the therapeutic effects of sea cucumber ovum hydrolysate (SCH) on premature ovarian failure (POF) and its underlying mechanism. We utilized a model of ICR mice induced with 100 mg/kg cyclophosphamide (CP) to evaluate the therapeutic influence of SCH on ovarian performance. The ovarian and uterine indices were significantly decreased in the POF group compared to the control group; however, these trends were notably reversed following SCH intervention. The therapeutic effects of SCH were positively reflected by the alterations induced by CP in levels of estradiol (E2), follicle-stimulating hormone (FSH), testosterone (T), luteinizing hormone (LH), and anti-Müllerian hormone (AMH). Regarding oxidative stress, SCH was found to enhance superoxide dismutase (SOD) activity and decrease malondialdehyde (MDA) levels, while also alleviating apoptosis in ovarian granulosa cells. Metabolomics analysis revealed hypoxanthine, mannitol, neocnidilide, tryptophan, palmitoleic acid, and protoporphyrinogen IX as potential biomarkers. In conclusion, SCH effectively improves POF induced by CP, thereby reinforcing the potential application of SCH in the domain of functional foods.

## 1. Introduction

Premature ovarian failure (POF), now more commonly referred to as primary ovarian insufficiency (POI), is a condition characterized by low estrogen levels in women prior to the age of 40 [1]. Estrogen, a vital sex hormone in the female body, is primarily secreted by the ovaries and plays a crucial role in various physiological processes. It significantly influences women’s reproductive health, bone health, metabolic regulation, and cardiovascular health [2,3]. The primary etiological factors associated with this syndrome include hysterectomy, genetic disorders such as Turner syndrome, cancer treatments, and autoimmune disorders [4]. The prevalence of this heterogeneous disease has been reported to range from 1% to 5%. Unfortunately, the disease is irreversible, and currently available management strategies, including hormone replacement therapy (HRT), can only alleviate the symptoms and side effects associated with the disease and related disorders [5,6].

In contemporary research, natural products play a significant role in the development of pharmaceuticals and food products. Their multi-pathway, multi-target mechanisms, combined with high efficacy and low toxicity, have been demonstrated in numerous studies to confer a wide array of beneficial activities, including anti-inflammatory, antioxidant, anti-proliferative, proapoptotic, and germ-protective effects [7]. The impact of various natural products (polyphenols, saponins, alkaloids, and polysaccharides) on ovarian function and POF has revealed their considerable potential [8]. Among these, flavonoids, also referred to as phytoestrogens, are non-steroidal polyphenolic compounds derived from plants. Their molecular structure bears similarities to endogenous estrogens and estradiol, which predisposes these phytoestrogens to bind to estrogen receptors across different cell types, thereby eliciting estrogenic or anti-estrogenic effects [9]. Recently, the influence of food-derived bioactive peptides on ovarian function has garnered attention. Reports indicated that oral administration of oyster peptide could restore the d-galactose-induced irregular estrous cycle and correct disturbances in serum levels of follicle-stimulating hormone (FSH) and luteinizing hormone (LH), while also reducing apoptosis in ovarian granulosa cells [10]. Furthermore, tilapia skin peptides have been shown to modulate the Bcl-2/Bax/caspase-3 apoptotic pathway and enhance the Nrf2/HO-1 signaling pathway, thereby improving POF by alleviating ovarian oxidative stress and reducing granulosa cell apoptosis [11].

Sea cucumbers, marine creatures widely distributed across the globe, have been utilized as tonics in Chinese culture [12,13]. They contain a variety of bioactive substances such as triterpene glycosides (saponins), chondroitin sulfate, glycosaminoglycans (GAGs), and peptides, which play crucial roles in numerous unique biological and pharmacological activities [14]. Recent studies have demonstrated that peptides derived from the sea cucumber (*Acaudina leucoprocta*) enhance sex hormone synthesis by upregulating the expression of StAR, Fshr and Cyp19a1 in the ovaries of mice with POF [15]. The potential of sea cucumbers to alleviate symptoms of POF is indicated by the presence of sex hormones (estradiol, progesterone, and testosterone) in their gonadal and neural tissues [16]. Sea cucumber ovum, a low-value by-product of industrial processing, represents a promising source of protein hydrolysates with multiple functional properties. Consequently, this study was designed to examine the mitigating effects of sea cucumber ovum hydrolysates (SCH) on POF. We examined and analyzed body weight, organ indices, estrous cycle, histopathology, hormone levels, antioxidant capacity and ovarian apoptosis. Additionally, metabolomics was employed to elucidate the potential mechanisms involved.

## 2. Materials and Methods

### 2.1. Reagents and Experimental Materials

The sea cucumber ovum used in this study was sourced from the Yantai Haizhongbao Seafood Trading Center (Yantai, China). Flavourzyme (15,000 U/g) was provided by Solarbio Biotechnology Co., Ltd. (Beijing, China). Cyclophosphamide (CP) was purchased from Shanghai Bichen Biochemical Technology Co., Ltd. (Shanghai, China), and soy isoflavones (SIF) was acquired from Shanghai Macklin Biochemical Technology Co., Ltd. (Shanghai, China). Superoxide dismutase (SOD) and malondialdehyde (MDA) kits were supplied by the Nanjing Jiancheng Bioengineering Institute (Nanjing, China). Enzyme-linked immunosorbent assay (ELISA) kits for testosterone (T), estradiol (E2), FSH, LH, and anti-Müllerian hormone (AMH) were sourced from Jiangsu Aikang Science & Technology Co., Ltd. (Suzhou, China). All other chemical reagents utilized in this study were of analytical grade and acquired from Sinopharm Chemical Reagent Co., Ltd. (Shanghai, China).

### 2.2. Preparation of SCH

The cleaned sea cucumber ovum was hydrolyzed at 50 °C using flavourzyme for a duration of 5 h. Following hydrolysis, the mixture was heated to 100 °C for 15 min to inactivate the enzyme. Upon chilling the sample with ice-cold water, the supernatant was achieved by centrifugation at 10,000 rpm for 15 min at 4 °C. Finally, the harvested supernatant was subjected to freeze-drying to yield the end product, SCH.

### 2.3. Determination of Dynamic Light Scattering (DLS)

SCH was dissolved in ultrapure water to create a 0.5 M solution, following a modification of the method proposed by Li et al. [17]. The ζ-potential, particle size, and polydispersity index (PDI) were accurately determined using the DLS technique (Nano Brook, Brookhaven, Nashua, NH, USA).

### 2.4. Determination of Amino Acids Composition

Modifications were implemented to determine the amino acid composition of SCH, in accordance with the protocol established by Chen et al. [18]. SCH was subjected to acid hydrolysis using 6 M hydrochloric acid at 110 °C for 22 h. The amino acid composition of SCH was then determined employing an L-8900 high-speed amino acid analyzer (Hitachi, Tokyo, Japan).

### 2.5. Animals and Experimental Design

Forty-five female ICR mice (8–10 weeks old, body weight 30 ± 2 g) were purchased from Jinan Pengyue experimental animal breeding Co., Ltd. (Jinan, China) and were maintained in a sterile environment with a 1-week acclimatization period.

After the acclimatization period, a POF model was established following the modifications of Luo et al. [15]. The 45 female mice were randomly divided into five groups: the control group, the model group (POF group), the positive control group (POF + SIF group), the POF + SCH low-dose group, and the POF + SCH high-dose group, with 9 mice in each group. Mice in the POF group received intraperitoneal injections of 100 mg/kg of CP for three consecutive days, while the other four groups were administered an equal volume of saline. Subsequently, the mice in the POF + SIF group received a daily gavage of 400 mg/kg SIF for four weeks. Meanwhile, those in the POF + SCH low-dose and high-dose groups were administered 200 mg/kg and 600 mg/kg SCH daily over a period of four weeks. Mice were monitored daily for body weight, and vaginal smears were collected for 8 days during both weeks 2 and 4.

### 2.6. Observation of Estrous Cycle

At weeks 2 and 4, the estrous cycle was divided into four distinct phases through cytological assessment of vaginal smears: pre-estrus, estrus, mid-pregnancy, and diestrus. During this period, saline was applied to 2–3 cm of the mouse vagina using a cotton swab, and the secretions were collected by gently rotating the swab. The collected samples were then smeared and stained with 0.4% methylene blue for observation and determination of the estrus stage under a light microscope.

### 2.7. Organ Index and Area of the Ovary

Following the experimental period, mice were euthanized by cervical dislocation, and the bilateral ovaries and uterus of the mice were collected and weighed. The area of the ovaries was calculated using ImageJ software (version 1.53c). Subsequently, the samples were either cryopreserved at −80 °C or preserved in 4% paraformaldehyde for further experiments.

### 2.8. Determination of Biochemical Parameters

Serum and ovarian tissue samples were collected and analyzed for SOD, MDA, T, E2, FSH, LH and AMH in accordance with the instructions provided with the assay kits.

### 2.9. Histological Observations on the Ovaries and Uterus

The uterus and ovaries were immersed in 4% paraformaldehyde for a fixation period of 24 h, followed by dehydration and embedding in paraffin. Tissue sections were cut to a thickness of 5 µm and stained with hematoxylin and eosin (H&E) prior to observation under a light microscope.

### 2.10. TUNEL

Ovarian paraffin sections were deparaffinized in water, and then the tissue was covered with a proteinase K working solution for 20 min. After rinsing twice with PBS, the sections were inactivated with 0.3% methanol peroxidase for 15 min. Subsequently, the sections were gently shaken to remove excess liquid, and buffer was applied dropwise in a circular manner. Following the removal of the equilibrium solution, the prepared TUNEL reaction solution was added dropwise, and coverslips were placed on top to allow for a 1 h reaction. Subsequently, the sections were washed in PBS three times for 5 min each, re-stained with DAPI for 15 min, and finally sealed. The apoptotic cells in the ovary were observed under a fluorescence microscope (Leica, Wetzlar, Germany).

### 2.11. LC-MS Based Untargeted Metabolomics Analysis

A total of 100 μL of serum sample was combined with 400 μL of extraction solution (MeOH:ACN, 1:1 (*v*/*v*)), which contained a deuterium internal standard, and vortexed for 30 s. The sample was then subjected to sonication in an ice-water bath for 10 min, followed by incubation at −40 °C for 1 h. Subsequently, the sample was centrifuged (12,000 rpm, 4 °C, 15 min), after which the supernatant was collected into a fresh glass vial for analysis. Quality control (QC) samples were generated through the combination of identical volumes from the sample’s upper liquid layer. The online assay was conducted using chromatographic separation of the target compounds on a Waters ACQUITY UPLC BEH Amide column (2.1 mm × 50 mm, 1.7 μm), with the ultra-high-performance liquid chromatograph being a Vanquish (Thermo Fisher Scientific, Waltham, MA, USA) ultra-high-performance liquid chromatograph (UHPLC). Liquid chromatography Phase A was composed of a water-based solution comprising 25 mM ammonium acetate and 25 mM ammonia, while Phase B consisted of acetonitrile. Subsequent data processing was conducted via the platform available at https://www.genescloud.cn/login (accessed on 18 May 2025).

### 2.12. Statistical Analysis

Statistical analyses were performed using GraphPad Prism 8 software, and results were expressed as mean ± SD. All tests were performed at least three times. Differences among various groups were assessed using the Tukey test in One-way ANOVA, with differences reported as *p* < 0.05.

## 3. Results

### 3.1. Structural Characterization of SCH

#### 3.1.1. ζ-Potential, Particle Size Distribution, and PDI of SCH

The ζ-potential, also known as the interfacial kinetic potential, is closely related to the stability of peptide solution systems. The results presented in Table 1 indicated that the ζ-potential of SCH was −37.11 ± 1.52 mV. This value suggests that the peptide molecule has a high proportion of acidic amino acids, and the electronegativity greater than −30 mV implies that the peptide molecules are unlikely to agglomerate due to strong repulsive forces, thereby contributing to the stability of the system. Furthermore, the particle size of SCH was measured at 465.17 ± 1.09 nm, with a PDI of 0.29 ± 0.01, further demonstrating that the SCH system possessed excellent physical stability.

#### 3.1.2. Amino Acid Composition of SCH

The results indicated that SCH contained 15 amino acids (Table 2). Among these, the negatively charged residues, specifically Glu and Asp, comprised 10.39% of the total, while the positively charged residues, including Arg, Lys, and His, accounted for 6.64%. This finding was consistent with the above reported negative ζ-potential. In addition, essential amino acids represented 16.91% of the total composition. Research indicates that arginine can enhance the ovarian antioxidant capacity during the luteal phase in ewes through the Nrf2/Keap1 signaling pathway [19]. Branched-chain amino acids (BCAAs), including Val, Leu, and Ile, serve as critical regulators of mammalian metabolic pathways and positively contribute to the treatment of ovarian diseases [20]. Consequently, SCH, which is rich in these amino acids, may have the potential to alleviate POF.

### 3.2. Body Weight Changes and Organ Indices in Mice

**Figure 1:**
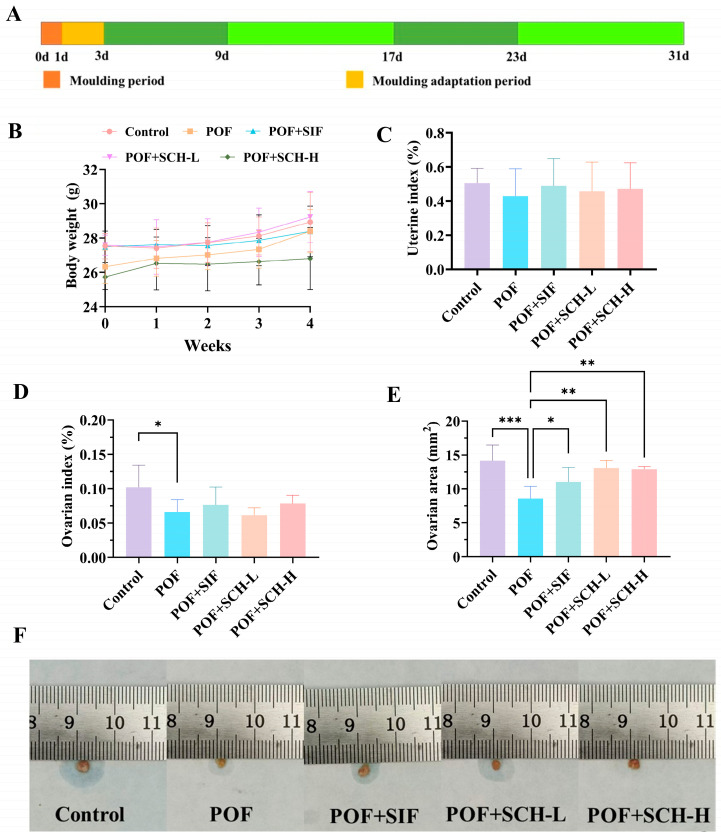
Experimental design, body weight, and organ indices. (**A**) Experimental design; (**B**) Body weight; (**C**) Uterine index; (**D**) Ovarian index; (**E**) Ovarian area; (**F**) Physical picture of ovaries. Statistical significance is indicated as follows: * *p* < 0.05, ** *p* < 0.01, and *** *p* < 0.001.

To evaluate the impact of SCH on CP-induced POF in mice, body weight changes and organ indices after 4 weeks of treatment were determined. The results of body weight, as depicted in Figure 1B, indicated no significant differences in SIF and SCH interventions when compared to the POF group. Similar findings were observed for uterine index (Figure 1C); although CP influenced the weight of the uterus in normal mice, this effect was not statistically significant. Furthermore, CP had a more pronounced impact on the ovaries, with a significantly lower ovarian index observed in the POF group compared to the control group (*p* < 0.05, Figure 1D). Notably, the results for ovarian area (Figure 1E) demonstrated that CP treatment significantly reduced the ovarian area (*p* < 0.001). Additionally, there was a significant increase in ovarian area among POF mice following interventions with SIF (*p* < 0.05), SCH-L (*p* < 0.01), and SCH-H (*p* < 0.01). These findings suggest a preliminary hypothesis that SCH may confer a protective effect on the ovaries of POF mice.

### 3.3. Effect of SCH on the Estrous Cycle in Mice with POF

**Figure 2:**
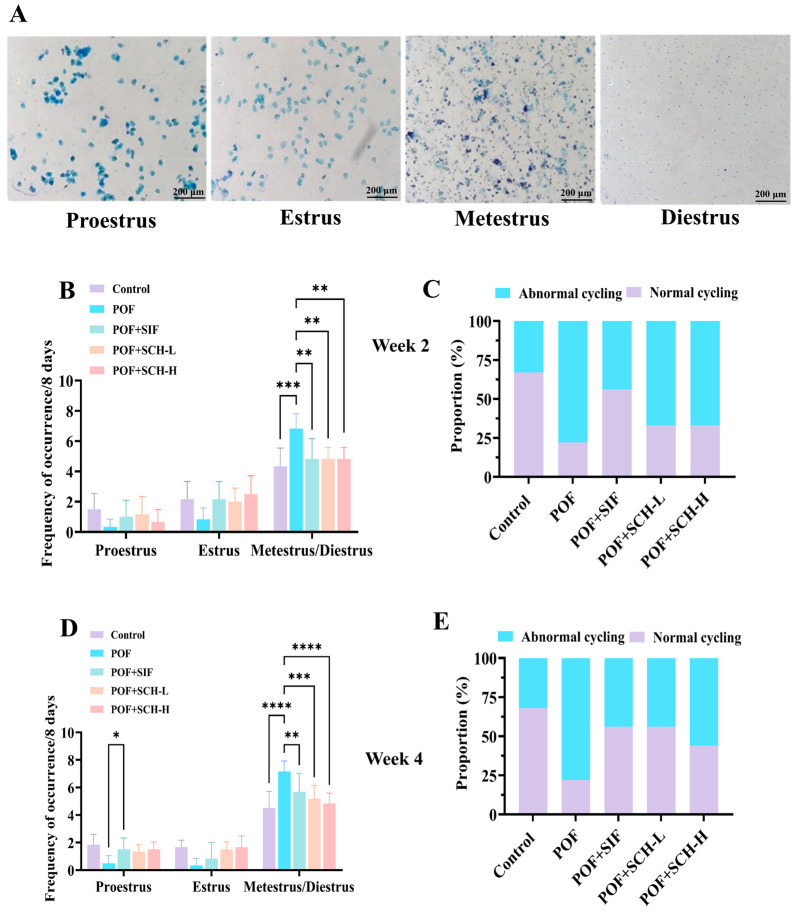
The effect of SCH on the estrous cycle. (**A**) Stained smears of the distinct phases of the estrous cycle: proestrus, estrus, metestrus, and diestrus; (**B**) Estrous cycle in POF model mice at week 2; (**C**) Percentage of cycling mice exhibiting normal estrous cycle at week 2; (**D**) Estrous cycle in POF model mice at week 4; (**E**) Percentage of cycling mice with normal estrous cycle at week 4. Data were expressed as mean ± SD (*n* = 9), with significance levels indicated as * *p* < 0.05, ** *p* < 0.01, *** *p* < 0.001, and **** *p* < 0.0001.

The impact of SCH on POF mice was assessed through vaginal smears to determine the estrus period in the subjects. The observation period was divided into two weeks: week 2 and week 4. During week 2, the estrous cycle status of the mice was first evaluated. In comparison to the control group, mice modeling POF exhibited a notable prolongation of metestrus and diestrus when exposed to CP (*p* < 0.001, Figure 2B). Conversely, a significant decrease in residence time was observed with the SIF, SCH-L, and SCH-H interventions (*p* < 0.01). Furthermore, the ability of the mice to complete an estrous cycle serves as an indicator of estrous cycle disorders. The proportion of mice that successfully completed an estrous cycle exhibited a marked reduction in the POF group relative to the control group. However, this proportion increased under the influence of SIF, SCH-L, and SCH-H, with SIF exhibiting the most pronounced effect (Figure 2C). In week 4, the effects of SIF (*p* < 0.01), SCH-L (*p* < 0.001), and SCH-H (*p* < 0.0001) on the residence time of POF mice during the metestrus and diestrus periods remained significant (Figure 2D). Notably, the proportion of mice capable of completing an estrous cycle increased in the SCH-L and SCH-H groups (Figure 2E).

### 3.4. Effect of SCH on Serum Sex Hormones in POF Mice

**Figure 3:**
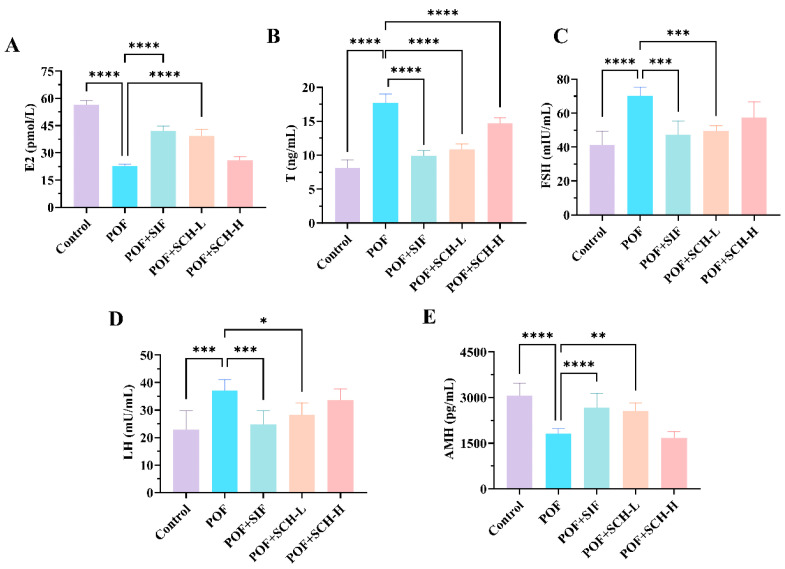
The effect of SCH on serum sex hormones. (**A**) E2; (**B**) T; (**C**) FSH; (**D**) LH; (**E**) AMH. Statistical significance is indicated as follows: * *p* < 0.05, ** *p* < 0.01, *** *p* < 0.001, and **** *p* < 0.0001.

Having established that SCH exerted a restorative effect on estrous cycle disorders in POF mice, we further investigated its impact on serum hormone levels in these animals. Based on the data presented in Figure 3, the POF group exhibited a significant reduction in the hormone levels of E2 and AMH compared to the control group (*p* < 0.0001, Figure 3A,E), while simultaneously showing a marked increase in the levels of T (*p* < 0.0001), FSH (*p* < 0.0001), and LH (*p* < 0.001) (Figure 3B–D). Notably, both SIF and SCH-L interventions demonstrated a positive trend in reversing these changes. Specifically, SIF and SCH-L significantly elevated E2 levels (*p* < 0.0001) while concurrently reducing T (*p* < 0.0001) and FSH (*p* < 0.001). In terms of LH and AMH, the overall effects were less pronounced compared to SIF, although SCH-L still exhibited significant impacts on both hormones. It is important to note that SCH-H, while significantly decreasing the elevated levels of T hormone resulting from CP (*p* < 0.0001), did not produce a significant, albeit somewhat reversible, effect on the other four indices.

### 3.5. Effect of SCH on Oxidative Stress

**Figure 4:**
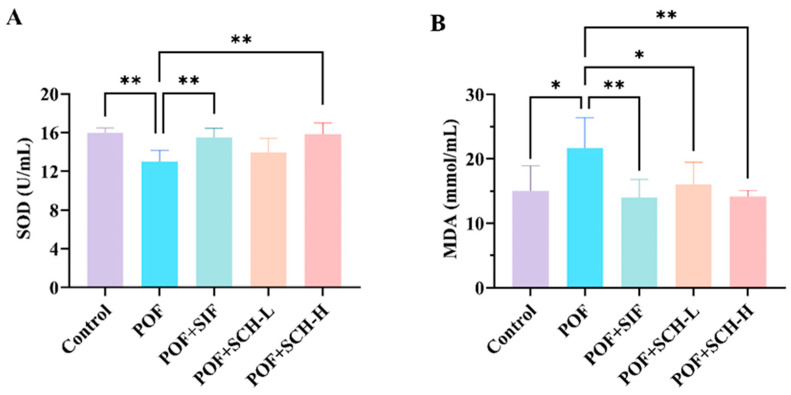
Oxidative stress levels in mouse serum. (**A**) SOD; (**B**) MDA. Statistical significance is indicated as follows: * *p* < 0.05, ** *p* < 0.01.

The activity of the SOD enzyme was significantly reduced in the POF group compared to the control group (*p* < 0.01), while MDA levels were considerably elevated (*p* < 0.05). Additionally, both SIF and SCH-H groups significantly restored SOD enzyme activity and reduced MDA levels in CP induced POF mice. However, the SCH-L group did not significantly elevate SOD enzyme activity (Figure 4A,B).

### 3.6. Histological Analysis

**Figure 5:**
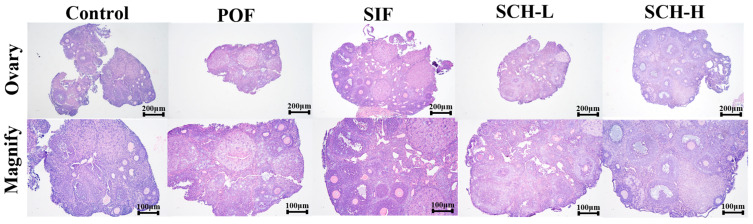
Histological analysis of ovaries.

As illustrated in Figure 5, the induction of CTX led to the development of follicles at all stages towards atretic follicles in the normal mouse ovary when compared to the control group. However, this effect was ameliorated by the effective intervention of SIF and SCH, which resulted in an increased number of healthy follicles at various stages of maturation and a reduction in atretic follicles. Additionally, there was minimal congestion and inflammation in the ovarian stroma, and the overall structure of the ovary was well preserved.

### 3.7. Impact of SCH on Ovarian Granulosa Cell Apoptosis in Mice with POF

**Figure 6:**
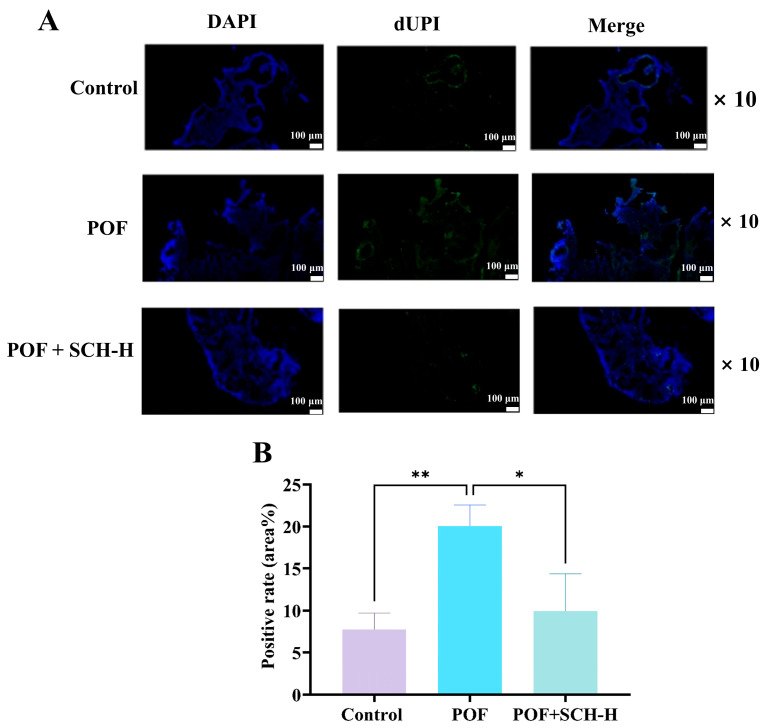
Effect of SCH on apoptosis of ovarian granulosa cells. (**A**) TUNEL staining to detect ovarian apoptosis (scale bar: 100 μm); (**B**) Apoptotic optical density of ovarian granulosa cells (*n* = 3). Statistical significance is denoted as follows: * *p* < 0.05 and ** *p* < 0.01.

The apoptosis of ovarian granulosa cells is a critical characteristic of POF. We investigated the impact of SCH on POF using TUNEL staining. The results indicated (Figure 6A,B) that POF significantly increased the apoptosis of ovarian granulosa cells compared to the control group (*p* < 0.01). However, apoptosis was notably reduced following SCH-H intervention (*p* < 0.05).

### 3.8. Effect of SCH on Serum Metabolic Profiles in POF Mice

**Figure 7:**
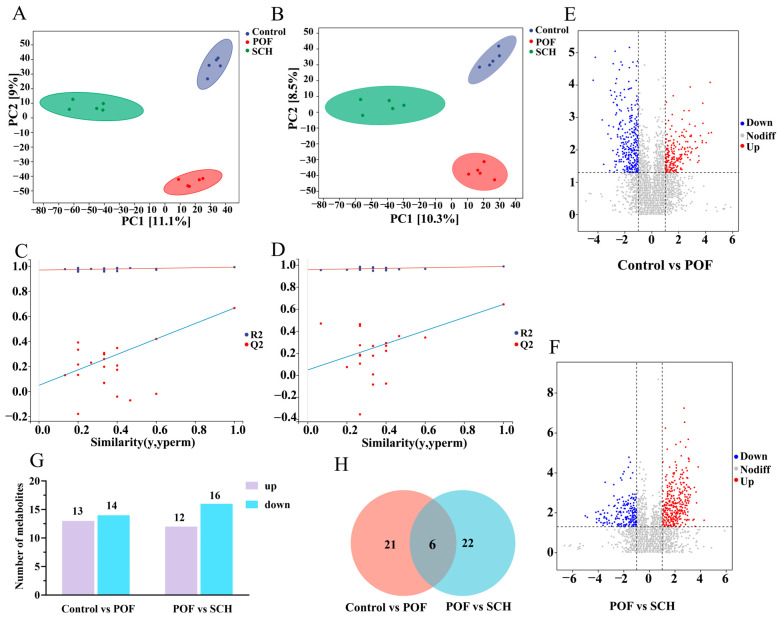
Effect of SCH on serum metabolic profiles in POF mice. (**A**,**B**) Positive ion modeling partial least squares-dispersion analysis (PLS-DA) and displacement test; (**C**,**D**) Negative ion modeling PLS-DA and displacement test. The red and blue lines represent the regression lines of R2 and Q2, respectively; (**E**,**F**) Univariate statistical analysis (Control vs. POF and POF vs. SCH); (**G**) Differential metabolite screening bar chart; (**H**) Venn diagram (Control vs. POF and POF vs. SCH).

**Figure 8:**
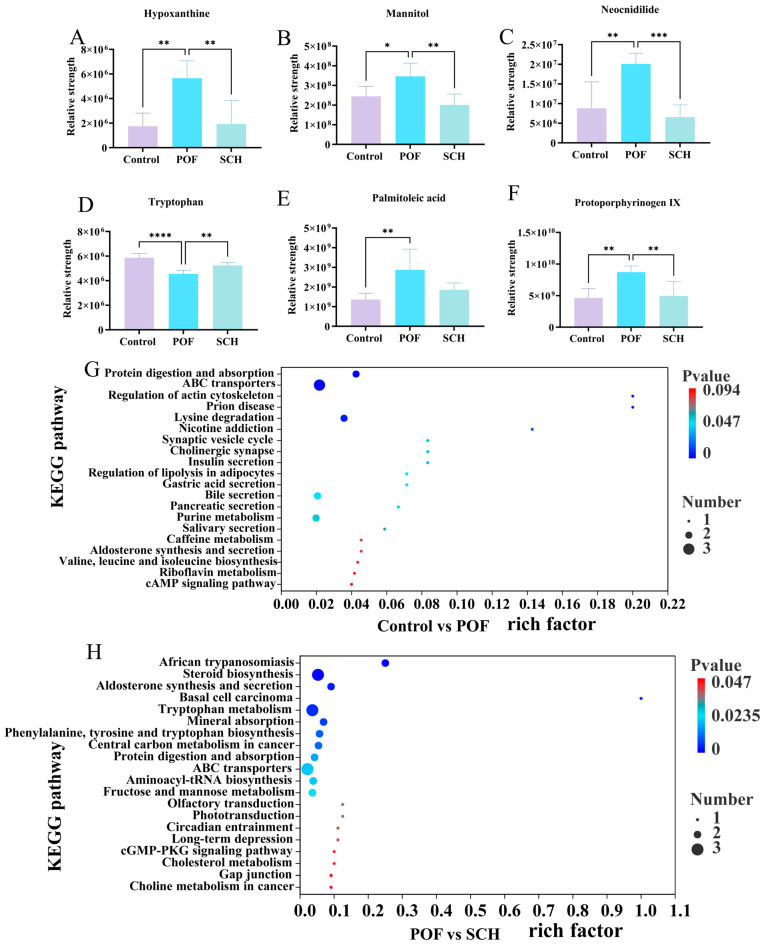
Effect of SCH on serum metabolites and metabolic pathways in POF mice. (**A**–**F**) Common differential metabolite; (**G**,**H**) Enrichment pathway (Control vs. POF and POF vs. SCH). Statistical significance is indicated as follows: * *p* < 0.05, ** *p* < 0.01, *** *p* < 0.001, and **** *p* < 0.001.

To elucidate the detailed mechanism of action of SCH concerning hormones, oxidative stress, and apoptosis, we analyzed serum metabolic profiles using UHPLC-Q-TOF-MS/MS to identify potential metabolites and metabolic pathways associated with POF. The results from the positive and negative partial least squares discriminant analysis model indicated (Figure 7A,B) that there was no overlapping region among the control, POF, and SCH groups, suggesting significant differences in metabolites across the three groups. Furthermore, the permutation test demonstrated (Figure 7C,D) that all Q2 points were lower than the original Q2 points on the far right, reinforcing the reliability and validity of our findings. Subsequently, univariate statistical analysis revealed differential metabolites between the control and POF groups, as well as between the POF and SCH groups (Figure 7E,F). Based on these findings, we screened differential substances for potential biomarkers using the criteria of *p* < 0.05 and VIP > 1. The results, illustrated in Figure 7G and Table 3, demonstrated that compared to the control group, 13 metabolites in the POF group were significantly up-regulated, while 14 metabolites were significantly down-regulated. Additionally, under the influence of SCH, 12 metabolites were significantly up-regulated and 16 metabolites were significantly down-regulated. Among these, eight metabolites associated with POF have been identified: hypoxanthine, mannitol, neocnidilide, tryptophan, 5-hydroxyindoleacetic acid, N6-acetyl-L-lysine, fisetin, and cyclic GMP. Additionally, mannitol, neocnidilide, tryptophan, 5-hydroxyindoleacetic acid, fisetin, and cyclic GMP may play a crucial role in preventing POF by inhibiting oxidative stress, repairing oocytes, preventing cell apoptosis, and regulating hormone levels. Notably, upon comparing the two data sets, we identified six significant metabolites common to all three groups (Figure 7H). Specifically, these metabolites included hypoxanthine, mannitol, neocnidilide, tryptophan, palmitoleic acid, and protoporphyrinogen IX (Figure 8A–F). Relative to the control group, the POF group demonstrated significant up-regulation of hypoxanthine, mannitol, neocnidilide, palmitoleic acid, and protoporphyrinogen IX, along with significant down-regulation of tryptophan. SCH treatment significantly reversed these trends.

Finally, differential abundance plots were employed to analyze the metabolic pathways across the three groups (Figure 8G,H). The POF group exhibited significant changes in protein digestion and absorption, ABC transporters, regulation of actin cytoskeleton, prion disease, and lysine degradation. Additionally, in POF mice under SCH treatment, significant alterations were noted in African trypanosomiasis, steroid biosynthesis, aldosterone synthesis and secretion, basal cell carcinoma, and the tryptophan metabolism.

## 4. Discussion

POF is a known side effect of cancer chemotherapy and is characterized as a multifactorial condition in which ovarian function declines in women under 40 years of age. This condition is primarily marked by elevated levels of gonadotropins, along with a decrease in ovarian reserve and estrogen levels [21,22]. In this study, sea cucumber ovum-derived SCH was prepared through enzymatic hydrolysis, and its therapeutic effects were evaluated in a CP-induced POF mouse model at doses of 200 mg/kg and 600 mg/kg. The modeling success with a dose of 100 mg/kg CP was confirmed based on the irregularities observed in the estrous cycle, alongside reductions in E2 levels and increases in FSH levels, as reported in previous studies on mice with POF.

An ototoxicity study assessing the effects of CP metabolites on cultured mouse ovaries revealed that metabolites such as 4-hydroxycyclophosphamide (4-HC) and phosphonamidite mustard (PM) exerted significant damaging effects on primordial and primary follicles [23]. AMH, a glycoprotein secreted by granulosa cells of pre-sinus and small sinus follicles in the ovary, serves as a crucial marker of follicular health [24]. In the initial stages of CP-induced ovarian damage, the number of small follicles diminishes rapidly due to their heightened sensitivity, resulting in a more pronounced decline in serum AMH levels compared to other sex hormones. As ovarian function deteriorates, the hypothalamic-pituitary-ovarian (HPO) axis stimulates the secretion of FSH and LH [25,26]. Within follicular membrane cells, 17α-hydroxylase and 17, 20-lyase facilitate T synthesis in response to LH [27]. The effects of SIF and SCH-L on the serum hormone alterations induced by CP in this study align with anticipated outcomes, and similar findings have been observed with tilapia skin peptides [11]. The suboptimal effect noted with SCH-H may be attributed to the higher dosage administered.

Studies have demonstrated that oxidative stress plays a critical role in the development of POF. In the present study, CP administration resulted in a decrease in SOD enzyme activity as well as an increase in MDA levels in mice, thereby elevating oxidative stress within the ovarian tissues. This condition led to heightened oxidative damage in follicular membrane cells and granulosa cells. Recent findings indicated that curculigoside (CUR) effectively reduced oxidative stress in ovarian tissue and provided protection against CP-induced ovarian injury [28]. Notably, SOD and MDA levels in this study showed significant positive results following SCH intervention, representing that SCH also played a protective role in ovarian health.

CP induces an imbalance between oxidative and antioxidant activities, resulting in the accumulation of reactive oxygen species (ROS) that can lead to ovarian damage through the production of oxygen free radicals, ultimately causing DNA damage and granulosa cell apoptosis [29]. The aberrant apoptosis of granulosa cells contributes to follicular failure, ultimately resulting in ovarian senescence or follicular atresia [30]. In this study, the TUNEL method was employed to detect ovarian granulosa cell apoptosis, which was significantly influenced by SCH intervention. Additionally, tilapia skin peptides were found to reduce ovarian granulosa cell apoptosis, aiding in the restoration of POF, and protein immunoblotting experiments demonstrated a significant increase in the anti-apoptotic protein Bcl-2, alongside the inhibition of Bax and caspase-3 expression, thereby offering protective effects for the ovary [11]. In addition, sea cucumber peptides derived from *Acaudina leucoprocta* did not significantly alter the relative expression of Bcl-2, Bax, and caspase-3 in response to POF [15]. Although these results were not examined in the present study, it is reasonable to speculate on their potential implications.

Furthermore, this study employed untargeted metabolomics to elucidate the potential mechanisms through which SCH protected ovarian function. We identified common differential metabolites present in three groups: hypoxanthine, mannitol, neocnidilide, tryptophan, palmitoleic acid, and protoporphyrinogen IX. SCH treatment can synergistically reshape the ovarian microenvironment across multiple dimensions, including abnormal cell apoptosis, oxidative stress imbalance, and hormonal metabolism disorders, through precise regulation of several key metabolites, thereby exerting therapeutic effects on POF. Notably, the level of hypoxanthine in the POF group was significantly higher than that in the control group (*p* < 0.01), which can inhibit granulocyte proliferation and induce apoptosis by disrupting the activity of MPF (cyclin B-CDK1 complex) within the PKA-Wee1/Myt1 signaling axis [31]. This is also associated with oxidative stress-induced cell death, exacerbating endothelial dysfunction and damaging the normal physiological state of ovarian cells [32]. However, following SCH intervention (Figure 8A), the level of hypoxanthine significantly decreased (*p* < 0.01), suggesting that it may improve CP-induced POF by promoting the degradation metabolism of hypoxanthine, such as by activating the reverse regulatory pathway of xanthine oxidase. Mannitol’s osmotic diuretic effect can not only reduce tissue edema, but also improve microcirculation and neutralize ROS, thereby minimizing cell and mitochondrial damage to the greatest extent possible [33]. Mice induced by CP may exhibit abnormally elevated drug metabolism due to the diuretic effect of mannitol in the regulation of their immune mechanisms (Figure 8B). Neocnidilide protects cells by inhibiting inflammatory mediators and reducing oxidative stress damage [34]. Figure 8D illustrated a significant increase in tryptophan levels following SCH intervention (*p* < 0.01). Serotonin, the primary metabolite of tryptophan [35], serves multiple roles as a neurotransmitter within the central nervous system, a blood factor, and a neurohormone that regulates peripheral organ function [36]. Furthermore, it influences the release of gonadotropins and regulates ovarian hormone levels.

In summarizing the differential metabolites presented in Table 3, alongside the six common differential metabolites, we identified that 5-hydroxyindoleacetic acid, N6-acetyl-L-lysine, fisetin, and cyclic GMP have specific effects on POF. Research indicates that the levels of 5-hydroxyindoleacetic acid influence ovarian cell function by regulating the hypothalamic-pituitary-ovarian (HPO) axis [37]. N6-acetyl-L-lysine, an acetylated form of lysine, exhibits changes in levels that are associated with protein modification and cellular stress, which in turn affects the epigenetic regulation of ovarian cells, leading to impaired follicular development [38]. Furthermore, studies demonstrate that fisetin activates the Sirt1 pathway in mouse oocytes through its antioxidant and anti-aging properties, thereby reducing oxidative stress and mitochondrial dysfunction, and delaying post-ovulation oocyte aging [39]. In the comparison between the POF and SCH groups, fisetin exhibited reduced activity, indicating that SCH intervention may mitigate oxidative stress damage to mouse ovarian germ cells. Additionally, cyclic GMP signaling is crucial for regulating follicular development and ovulation; its dysregulation may exacerbate granulosa cell apoptosis, consequently promoting follicular atresia [40].

In summary, SCH modulates CP-induced metabolic alterations in mice. Notably, KEGG enrichment analysis indicates that the tryptophan and cyclic GMP pathways in SCH metabolism represent the core mechanisms underlying CP pathogenicity in normal mice. Furthermore, the steroid biosynthesis pathway serves as a fundamental route for ovarian hormone synthesis. By promoting the release of androstenedione, testosterone, and dehydroepiandrosterone sulfate (DHEAS), it alleviates the functional decline of ovarian granulosa cells and theca cells in individuals with POI [41]. In the metabolic pathways of CP, protein digestion and absorption, as well as the ABC transporter and lysine degradation, are significantly enriched. Studies have demonstrated that lysine succinylation is a conserved and novel post-translational modification (PTM) that can influence protease activity and gene expression [42]. Furthermore, succinylated lysine is highly enriched in mitochondrial (Mt) proteins and extra-Mt proteins that regulate cell proliferation, oxidative damage, and apoptosis [43,44,45]. Le et al. [46] confirmed that lysine succinylation levels in POI mouse ovaries are higher than those in normal ovaries, suggesting that elevated lysine succinylation levels may inhibit ovarian reproductive and endocrine functions.

## 5. Conclusions

In conclusion, the findings of this study demonstrated that the oral administration of SCH could modulate serum metabolites to improve POF in mice. Additionally, lower doses of SCH demonstrated more favorable effects regarding hormonal levels and oxidative stress outcomes; however, the specific functions of SCH require further clinical research. Consequently, more rigorous and extensive studies should be warranted before SCH could be considered for the development of functional foods in the future. The findings of this study are significant for the regulation of sex hormones and will contribute to the advancement of marine functional foods.

## Figures and Tables

**Table 1:** ζ-potential, particle size distribution, and PDI of SCH.

ζ-Potential (mV)	Particle Size Distribution (nm)	PDI
SCH −37.11 ± 1.52	465.17 ± 1.09	0.29 ± 0.01

**Table 2:** The amino acid composition of SCH.

Amino Acid	Abbreviation	Ratio (g/100 g)
Glutamic acid #	Glu	6.29
Aspartic acid #	Asp	4.10
Leucine *	Leu	3.12
Valine *	Val	2.93
Arginine #	Arg	2.90
Lysine *	Lys	2.76
Alanine #	Ala	2.65
Threonine *	Thr	2.38
Glycine #	Gly	2.18
Serine #	Ser	2.05
Isoleucine *	Ile	2.04
Phenylalanine *	Phe	1.70
Tyrosine #	Tyr	1.12
Methionine *	Met	1.00
Histidine *	His	0.98
Acidic amino acids		10.39
Basic amino acids		6.64
Essential amino acids *		16.91
Non-essential amino acids #		21.29
Total amino acids		38.20

Amino acids marked with * indicate essential amino acids, while those marked with # represent non-essential amino acids.

**Table 3:** The differential metabolite screening.

Group	Name	Regulation
Control vs. POF	Glutarate semialdehyde	Up
	Mesaconate	Down
	L-Isoleucine	Up
	Acetylcholine	Down
	p-Synephrine	Up
	Xanthine	Down
	4-Quinolinecarboxylic acid	Up
	N6-Acetyl-L-lysine	Down
	5-Hydroxyindoleacetic acid	Up
	Galactaric acid	Down
	L-Cystine	Down
	Lumichrome	Down
	Pentostatin	Down
	N2-Malonyl-D-tryptophan	Down
	3-Ketosphingosine	Down
	Arachidic acid	Down
	Corticosterone	Down
	Deoxycholic acid	Up
	7a-Hydroxy-5b-cholestan-3-one	Up
	LysoPA(16−0−0−0)	Down
	5-Oxoavermectin “2a” aglycone	Up
	Hypoxanthine	Up
	Mannitol	Up
	Neocnidilide	Up
	Tryptophan	Down
	Palmitoleic acid	Up
	Protoporphyrinogen IX	Up
POF vs. SCH	Hypoxanthine	Down
	Mannitol	Down
	Neocnidilide	Down
	Tryptophan	Up
	Palmitoleic acid	Down
	Protoporphyrinogen IX	Down
	Methylmalonic acid	Down
	Acetylcysteine	Up
	L-Phenylalanine	Up
	3,4-Dihydroxyphenylpropanoate	Down
	N-Formyl-L-methionine	Up
	D-Fructose	Up
	Sequoyitol	Down
	L-Tryptophan	Down
	L-Kynurenine	Up
	Methyldopa	Down
	N-Acetylserotonin	Up
	gamma-Glutamylalanine	Down
	Apiole	Down
	N2-Succinyl-L-ornithine	Up
	Glycerophosphocholine	Down
	Octadecanamide	Up
	Fisetin	Down
	Cyclic GMP	Up
	Cholesterol	Up
	Zymosterol intermediate 2	Up
	24,25-Dihydrolanosterol	Down
	L-Olivosyl-oleandolide	Down

## Data Availability

The data presented in this study are available on request from the corresponding author.

## Abbreviations

The following abbreviations are used in this manuscript:

SCH	Sea cucumber ovum hydrolysate
POF	Premature ovarian failure
CP	Cyclophosphamide
E2	Estradiol
FSH	Follicle-stimulating hormone
T	Testosterone
LH	Luteinizing hormone
AMH	Anti-Müllerian hormone
SOD	Superoxide dismutase
MDA	Malondialdehyde
POI	Primary ovarian insufficiency
SIF	Soy isoflavones
ELISA	Enzyme-linked immunosorbent assay
HRT	Hormone replacement therapy
GAGs	Glycosaminoglycans
DLS	Determination of dynamic light scattering
PDI	Polydispersity index
H&E	Hematoxylin and eosin
QC	Quality control
UHPLC	Ultra-high-performance liquid chromatograph
ANOVA	One-way analysis of variance
